# Protective effect of hydrogen-saturated saline on acute lung injury induced by oleic acid in rats

**DOI:** 10.1186/s13018-017-0633-9

**Published:** 2017-09-19

**Authors:** Youguo Ying, Haizhou Xu, Min Yao, Zonghe Qin

**Affiliations:** 1 Departments of Intensive Care Unit, Shanghai 9th People’s Hospital, Shanghai Jiao Tong University School of Medicine, 280 Mo He Rd, Shanghai, 201999 People’s Republic of China; 2 Department of Emergency, Changhai Hospital, Second Military Medical University, Shanghai, 200433 People’s Republic of China

**Keywords:** Hydrogen-saturated saline, Lung injury, Oxidative stress, Inflammatory factors

## Abstract

**Background:**

The purpose of the study is to investigate the role and mechanisms of hydrogen-saturated saline (HSS) in the acute lung injury (ALI) induced by oleic acid (OA) in rats.

**Methods:**

Rats were treated with OA (0.1 mL/kg) to induce ALI and then administered with HSS (5 mL/kg) by intravenous (iv) and intraperitoneal (ip) injection, respectively. Three hours after the injection with OA, the arterial oxygen partial pressure (PaO_2_), arterial oxygen saturation (SaO_2_), carbon dioxide partial pressure (PaCO_2_), and bicarbonate (HCO_3_^−^) levels were analyzed using blood gas analyzer. In addition, the levels of malondialdehyde (MDA), tumor necrosis factor-α (TNF-α), and interleukin 1β (IL-1β) and myeloperoxidase (MPO) activity were measured by commercial kits, and pathological changes of lung tissue were examined by HE staining. Finally, the correlations of MPO activity or MDA level with the levels of TNF-α or IL-1β were analyzed by Pearson’s correlation analysis.

**Results:**

We found decreased PaO_2_ levels and the pathological changes of lung tissue of ALI after OA injection. In addition, OA increased the levels of MDA, TNF-α, and IL-1β, as well as MPO activity in lung tissues (*P* < 0.05). However, after treatment with HSS, all of these changes were alleviated (*P* < 0.05), and these changes were mitigated when treated with HSS by ip then iv injection (*P* < 0.05). Furthermore, MDA level and MPO activity were positively correlated with TNF-α and IL-1β levels in the lung tissue, respectively (*P* < 0.01).

**Conclusion:**

HSS attenuated ALI induced by OA in rats and might protect against ALI through selective resistance to oxidation and inhibiting inflammatory infiltration.

## Background

Acute lung injury (ALI) is a common complication with acute hypoxemic respiratory failure and systemic inflammatory, which is characterized by hypoxemia, pulmonary infiltrates, and edema [1]. In the USA, the prevalence of ALI increases from 2% in 1988 to 22% in 2008 in hospital admissions [2]. ALI can be induced by a variety of risk factors such as sepsis, multiple traumatic injuries, cardiopulmonary bypass, pneumonia, and swine flu [1]. Although our understanding for the pathogenesis, treatment, and long-term outcomes of ALI has significantly improved, the process and prognosis related to ALI involved still cannot be accurately judged [3]. Thus, it is essential to search for more effective treatment methods.

The occurrence and development of ALI are tightly related to the activations of macrophages and pulmonary endothelium, which induce the production of various cytotoxic and pro-inflammatory compounds, including reactive oxygen species, proteolytic enzymes, nitrogen species, lipid mediators, cationic proteins, and inflammatory cytokines [4]. Recently, several studies have demonstrated a vital role of oxidative stress in the pathogenesis of ALI [5–8]. Noteworthily, it is well known that oleic acid (OA) can induce ALI in animals [9]. Previous study has shown that OA is associated with oxidative stress through inducing increased contents of lactate dehydrogenase and lipid peroxidation products in the bronchial alveolar lavage fluid (BALF) of guinea pigs, as well as decreased glutathione/glutathione disulfide [9]. Moreover, pretreatment with catalase and superoxide dismutase attenuates both the generation of oxygen radicals and the pulmonary edema triggered by OA [10, 11], suggesting that oxidative stress is involved in OA-induced ALI.

Currently, therapeutic strategies for ALI have focused on limiting oxidative lung injury [5]. Hydrogen, a colorless, odorless, and oxidation-resistant molecule, can protect DNA against oxidative damage by selectively removing hydroxyl free radicals and peroxynitrate, as well as inhibiting inflammation [12, 13]. It is reported that hydrogen can alleviate many organ damages, including the heart, lung, brain, liver, kidney, and intestine [14]. Moreover, breathing 2% hydrogen for 6 h is beneficial in patients with severe sepsis caused by multiple organ failure [15]. Recently, hydrogen-saturated saline (HSS) is widely proved to exert a therapeutic effect on many diseases such as sepsis, oxygen toxicity, ischemia-reperfusion (I/R) injury, stroke, multiple organ dysfunction syndrome, and neurodegenerative diseases [14]. HSS can improve mild cerebral ischemia-induced anoxic damage by intraperitoneal (ip) or intravenous (iv) injection with accurate dosage in newborn mice [16]. In addition, HSS injection has recently been found to protect against ALI caused by scalding [17]. However, the role and mechanism of HSS in OA-induced ALI in rats is still unclear.

In the present study, we treated OA-induced ALI rats with HSS and then analyzed arterial blood gas. Because the pathogenesis of ALI was associated with oxidative damage and inflammation, we further detected the levels of malondialdehyde (MDA) and myeloperoxidase (MPO), as well as inflammatory factors such as interleukin 1β (IL-1β) and tumor necrosis factor-α (TNF-α). MDA is the terminal product of unsaturated fatty acid peroxidation and considered as a biological marker of oxidative stress [18]. In addition, MPO, a major neutrophil enzyme, can reflect the extent of neutrophil infiltration, which is taken as an evaluation index of lung injury [19]. This study aimed to explore the role and the related mechanisms of HSS in OA-induced ALI rats.

## Methods

### Animals

A total of 40 Sprague-Dawley rats (adult male, 250–300 g) were provided by the Experimental Animal Center of the Chinese Academy of Sciences and acclimatized under appropriate condition (natural day-night cycle and free access to food and water) for a week before the trial. Approval from the Institutional Animal Care and Use Committee of Secondary Military Medical University, ROC, was obtained prior to using the animals for research.

### Reagents and equipment

HSS was kindly provided by Professor Sun Xuejun of the Secondary Military Medical University, ROC. Solutions were freshly prepared every week and maintained at 4 °C to ensure a stable hydrogen concentration of 0.6 mM. High-purity OA was purchased from Sigma (St. Louis, MO, USA). MDA and MPO assay kits were obtained from the Nanjing Jiancheng Bioengineering Institute, China. Enzyme-linked immunosorbent assay (ELISA) kits for TNF-α and IL-1β were provided by Peprotech (NJ, USA). Equipment in this study included MK3 microplate reader (Thermo-Scientific, MC, USA), MK2 microplate washer (Thermo-Scientific, MC, USA), electro-heating standing-temperature cultivator (Shanghai Yuejin Medical Instruments Factory, China), TGL-168 centrifuge (Shanghai Anting Scientific Instrument Factory, China), SmartSpec Plus spectrophotometer (Bio-Rad, Hercules, CA, USA), micro-electric tissue homogenizer (Kimble, USA), GM3000 blood gas analyzer (Instrumentation Laboratory, MA, USA), and light microscopy (Olympus, Japan).

### Experimental design

Rats were fasted for 12 h before experiments, but given free access to drinking water and allowed to breathe normally during the experimental process. Forty rats were randomly and equally divided into four groups: control group (*n* = 10), ALI group (*n* = 10), ALI + HSS (iv) group (*n* = 10), and ALI + HSS (ip) group (*n* = 10). Rats in the control group received an injection of saline (0.1 mL/kg) via the tail vein, rats in the ALI group received a similar iv injection of high-purity OA dissolved in saline (0.1 mL/kg), rats in the ALI + HSS (iv) group received the equal dose of OA by iv injection as rats in the ALI group and then an injection of HSS (5 mL/kg) via the tail vein 5 min after the injection of OA, and rats in the ALI + HSS (ip) group received the equal dose of OA by iv injection as rats in the ALI group and then received an ip injection of HSS (5 mL/kg) 5 min after the OA injection. Three hours after the OA injection [20], the animals in the four groups were anesthetized with 3% pentobarbital sodium (0.2 mL/100 g, ip), laid supine, and then fastened to collect arterial blood. All animals survived for all experiments. After the arterial blood was collected, the rats in all the groups were sacrificed by spinal dislocation under anesthesia. The left lungs were collected for histopathological analysis. The right lungs were taken for the detection of MDA, MPO, TNF-α, and IL-1β.

### Arterial blood gas analysis

We collected arterial blood from the right femoral artery after the OA injection. For arterial blood collection, an indwelling IV catheter was inserted into the right femoral artery, and a physiological recorder was connected to monitor blood pressure and heartbeat. Arterial blood gas analysis was conducted after blood pressure had been stable for 5 min. The arterial oxygen partial pressure (PaO_2_), arterial oxygen saturation (SaO_2_), carbon dioxide partial pressure (PaCO_2_), and bicarbonate (HCO_3_^−^) levels were analyzed using a blood gas analyzer [21].

### Hematoxylin and eosin staining

Three hours after the OA injection, the left lung tissues were post-fixed in 4% formaldehyde buffer for 48 h and then paraffin-embedded tissue were sliced into 5-μm-thick sections. Next, sections were dehydrated, stained with hematoxylin and eosin, dehydrated, cleared, and mounted with neutral resin. Light microscopy was used to observe the sections.

### Detection of MDA and MPO in the lung

Three hours after the OA injection, the right lungs were dissected, sheared, rinsed with 4 °C saline to remove blood, and then filter dried. Lung tissue homogenates were prepared using a micro-electric tissue homogenizer. The tissue homogenates were used to measure MDA concentration and MPO activity according to the corresponding manufacturer’s instructions. For MDA detection, the tissue homogenates were mixed with the reaction reagents (MDA assay kit, Nanjing Jiancheng Bioengineering Institute) and incubated for 40 min at 95 °C. After centrifugation at 3500 r/min for 10 min, the supernatant was used to calculate MPO activity according to maximal absorbance at 532 nm. For MPO detection, the tissue homogenates were mixed with reagent II at a ratio of 1:1 (MPO assay kit, Nanjing Jiancheng Bioengineering Institute), followed by the addition of reagent IV and chromogenic agent and reaction for 10 min at 60 °C. Lastly, MPO activity was calculated according to maximal absorbance at 460 nm.

### Detection of TNF-α and IL-1β in the lung

Three hours after the OA injection, the right lungs were dissected, sheared, rinsed with 4 °C saline to remove blood, and then filter dried. The contents of TNF-α and IL-1β in the right lungs were detected using commercial enzyme-linked immunosorbent assay (ELISA) kits following the manufacturer’s instruction. Briefly, lung tissue homogenates were prepared using a micro-electric tissue homogenizer and then mixed with saline at a ratio of 1:9 (10%). Next, 10% homogenates were added into each well of a 96-well plate with coating antigen. After incubation for 2 h at 37 °C, the 96-well plate was washed, blocked, and then incubated with antibody for 1 h at 37 °C. Subsequently, after washing for three times, the plate was added with a second antibody for 1 h and then incubated with the substrate solution for 30 min at 37 °C. Eventually, concentrations of TNF-α and IL-1β were calculated according to maximal absorbance at 450 nm.

### Statistical analysis

SPSS 13.0 software (SPSS Inc., Chicago, IL, USA) was used for statistical analysis. All measured values are presented as mean ± standard deviation (SD). Variables between groups in normal distribution were analyzed using one-way analysis of variance followed by the least significant difference test (LSD), and variables between groups in non-normal distribution were analyzed using the Kruskal-Wallis test. Comparison between the two groups was performed using the *t* test. The correlations of MPO activity or MDA level with the levels of TNF-α or IL-1β were analyzed by Pearson’s correlation analysis. A *P* value of less than 0.05 was considered statistically significant.

## Results

### Arterial blood gas analysis

Compared with the control group, PaO_2_ levels in the ALI, ALI + HSS (iv), and ALI + HSS (ip) groups were significantly decreased 3 h after OA injection (*P* < 0.05) (Table 1). In addition, PaO_2_ in the ALI + HSS (iv) and ALI + HSS (ip) groups were higher than that in the ALI group (*P* < 0.05), and PaO_2_ in the ALI + HSS (ip) group was higher than that in the ALI + HSS (iv) group (*P* < 0.05) (Table 1). However, there were no significant differences in SaO_2_, PaCO_2_, and HCO_3_^−^ levels among the four groups (Table 1).

**Table 1:** Arterial blood gas analysis results in the control, ALI, ALI + HSS (iv), and ALI + HSS (ip) groups (mean ± SD)

Index	Control	ALI	ALI + HSS (iv)	ALI + HSS (ip)
PaO_2_ (mmHg)	92.80 ± 4.36	72.46 ± 4.86^*^	81.93 ± 1.87^*#^	85.80 ± 1.91^*#△^
SaO_2_ (%)	95.80 ± 0.72	96.70 ± 1.58	94.28 ± 0.35	94.70 ± 0.48
PaCO_2_ (mmHg)	45.81 ± 1.73	37.20 ± 2.55	38.98 ± 2.47	39.72 ± 1.52
HCO_3_^−^ (mmol/L)	28.88 ± 0.89	28.20 ± 3.82	27.34 ± 1.06	27.22 ± 1.01

*ALI* acute lung injury, *HSS* hydrogen-saturated saline, *PaO*_*2*_ arterial oxygen partial pressure, *SaO*_*2*_ arterial oxygen saturation, *PaCO*_*2*_ carbon dioxide partial pressure, *HCO*_*3*_^*−*^ bicarbonate

Compared with control group, *P < 0.05; compared with ALI group, ^#^ *P* < 0.05; compared with ALI + HSS (iv) group, ^△^ *P* < 0.05

### Lung tissue histopathological changes

**Fig. 1:**
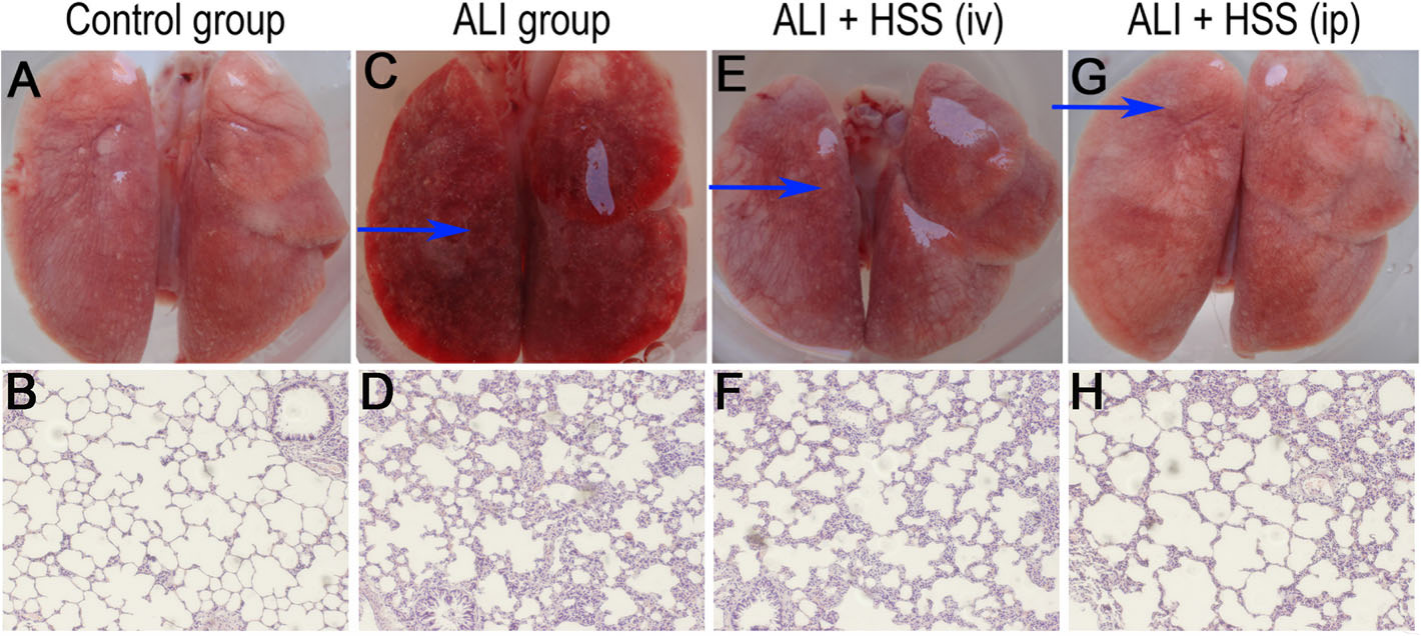
Visual observation and histopathological examination of lung tissue in the control, ALI, ALI + HSS (iv), and ALI + HSS (ip) groups (*n* = 10 in each group). Compared with the control group (**a**, **b**), rat lung tissue surface was rough and bleeding under the naked eye (**c**) and serious injury was observed under optical microscope (**d**) in the ALI group. However, after treatment with HSS, rat lung tissue surface showed milder smooth and pink pulmonary tissue surface under the naked eye (**e**, **g**) and lung injury had a substantial improvement under optical microscope (**f**, **h**) in the ALI + HSS (iv) and ALI + HSS (ip) groups, and all of these changes were alleviated in the ALI + HSS (ip) group rats than in the ALI + HSS (iv) group rats. Original magnification; HE staining × 100. The arrowheads point to the bleeding area. ALI acute lung injury, HSS hydrogen-saturated saline

In the control group, the pulmonary tissue surface of the rats was pink and smooth and no leakage or bleeding was visible under the naked eye (Fig. 1a). Consistently, hematoxylin and eosin (HE) staining showed clear alveolar structure and thin alveolar walls, and no exudate from alveoli could be found under the optical microscope (Fig. 1b). In contrast, under the naked eye, rats’ pulmonary tissue surface in the ALI group was rough and bleeding (Fig. 1c), and HE staining also showed serious injury in lung tissues, including pulmonary tissue hyperemia and patchy necrosis, pulmonary interstitial edema, diffuse hemorrhage, and influx of inflammatory cells (Fig. 1d). After treatment with HSS, the rats in the ALI + HSS (iv) and ALI + HSS (ip) groups showed milder smooth and pink pulmonary tissue surface under the naked eye (Fig. 1e, g), as well as milder pulmonary edema, inflammatory cell infiltration, and alveolar hemorrhage under the microscope (Fig. 1f, h), and all of these changes were alleviated in the ALI + HSS (ip) group rats than those in the ALI + HSS (iv) group rats (Fig. 1e, g, f, and h).

### Changes of MDA, MPO, TNF-α, and IL-1β in the lung

**Fig. 2:**
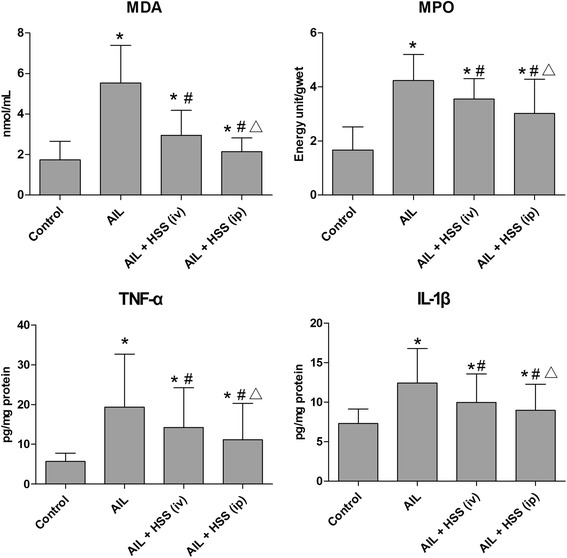
Changes of lung MDA, MPO, TNF-α, and IL-1β in the control, ALI, ALI + HSS (iv), and ALI + HSS (ip) groups (*n* = 10 in each group). MDA, TNF-α, and IL-1β levels as well as MPO activity were significantly increased in the ALI, ALI + HSS (iv), and ALI + HSS (ip) groups compared with the control group. However, HSS significantly decreased the levels of MDA, TNF-α, and IL-1β and the activity of MPO of lung tissues compared with the ALI group, and all of these measurements were lower in the ALI + HSS (ip) group than in the ALI + HSS (iv) group. Compared with the control group, *P < 0.05; compared with the ALI group, ^#^ *P* < 0.05; compared with the ALI + HSS (iv) group, ^△^ *P* < 0.05. ALI acute lung injury, HSS hydrogen-saturated saline, MDA malondialdehyde, MPO myeloperoxidase, TNF-α tumor necrosis factor-α, IL-1β interleukin 1β

Compared with the control group, MDA, TNF-α, and IL-1β levels as well as MPO activity were significantly increased (*P* < 0.05) in the ALI, ALI + HSS (iv), and ALI + HSS (ip) groups (Fig. 2). The levels of MDA, TNF-α, and IL-1β as well as the activity of MPO of lung tissues were significantly decreased in the ALI + HSS (iv) and ALI + HSS (ip) groups compared with the ALI group (*P* < 0.05), and all of these measurements were lower in the ALI + HSS (ip) group than in the ALI + HSS (iv) group (*P* < 0.05) (Fig. 2).

### Correlation analysis

Pearson’s correlation analysis showed that MDA level was positively correlated with TNF-α (*r* = 0.854, *P* = 0.007) and IL-1β levels (*r* = 0.822, *P* = 0.012). Similarly, MPO activity also had positive correlations with TNF-α (*r* = 0.873, *P* = 0.005) and IL-1β levels (*r* = 0.804, *P* = 0.016).

## Discussion

ALI was considered as a long-term illness and increased the burden on the individual patients as well as society [1]. It was urgent to search for an effective treatment for ALI. In the present study, we examined the effects of HSS on oxidative damage and inflammatory pathological changes in OA-induced ALI rats. Our study found that arterial PaO_2_ significantly decreased in OA-induced ALI rats and lung tissues emerged with hyperemia and patchy necrosis, pulmonary interstitial edema, diffuse hemorrhage, and influx of inflammatory cells. Hypoxemia is a main clinical characteristic and a major cause of death in patients with ALI [4]. Common symptoms of hypoxemia were impaired alveolar cells, widened alveolar septum, severe pulmonary congestion and edema, and alveolar collapse [4, 22]. These results indicated that OA effectively induced ALI in the rat model. With the intervention of HSS, arterial PaO_2_ increased, and histopathological damages of the lung, including pulmonary edema, inflammatory cell infiltration, and alveolar hemorrhage, were significantly alleviated, indicating that HSS had an improvement role in OA-induced ALI rats.

Noteworthily, we found that injection of HSS by ip had a more effective therapy on OA-induced ALI than injection of HSS by iv. Researchers in Japan found that continuous administration (8 h/d) of small doses (0.08 ppm) of 1/20-saturated hydrogen water (1.5 ppm) had significant therapeutic effects on MPTP-induced Parkinson’s disease in mouse [23], indicating that the low doses of hydrogen were more appropriate for effective treatment. In addition, most studies chose an ip injection of HSS [24–26]. Previous study had shown that compared with iv injection (half-lives 20.4 h), liposomes with ip administration appeared in the blood with half-lives (0.6 h) [27] because ip injection had a stronger absorption ability and could maintain the effective concentration of an injected drug for a long time, which might achieve the better effects even with a low drug concentration than iv injection with larger doses. Thus, continuous ip delivery of drugs at low doses might be the most suitable way to maximize the drug’s effect.

To further explore the mechanisms of the protective effect of HSS on OA-induced ALI, we detected the level of MDA. It was well accepted that oxidative stress was an important mechanism in OA-induced ALI [10]. Our study found that the level of MDA in lung tissue significantly increased; however, MDA level reduced after treatment with HSS. Consistent with our results, Shi et al. [24] also suggested that HSS injected by ip could reduce MDA content in rats with acute lung I/R injury. These results indicated that HSS could at least partly perform an anti-oxidant function in OA-induced ALI. This might be explained by the ability of hydrogen to remove hydroxyl radicals (OH) with strong cytotoxicity, reduce lipid peroxidation, and increase cell vitality [28].

In addition, we also found that OA could induce increased activity of MPO, which could be mitigated by HSS. Similarly, after treatment with HSS by caudal vein injection, the MPO activity dramatically reduced the liver injury induced by lipopolysaccharides [29]. This result indicated that HSS could reduce lung injury in rats caused by OA, possibly through the inhibition of inflammatory cell infiltration in the lung tissue [30]. Furthermore, according to our results, HSS could significantly attenuate the levels of TNF-α and IL-1β in lung tissues. Previous studies had demonstrated that the occurrence of the lung injury was related to the increased levels of TNF-α and IL-1β, which synergized and enlarged the systemic inflammation reaction [31, 32]. TNF-α were often synergistically functioned with IL-1β [33], which could stimulate the production and release of a variety of inflammatory cells, trigger the release of oxygen free radicals and lipid metabolites, and then cause damage of the alveolar surface active material and endothelial cells, as well as blood capillary leakage, eventually leading to ALI [34]. Consistent with our results, it had been demonstrated that HSS injected by ip could reduce the contents of TNF-α and IL-1β in hyperoxic lung injury [30]. Therefore, HSS might exert its protective effect against lung tissue injury by inhibiting the inflammatory cascade.

Our analyses moreover found that MDA level and MPO activity were significantly correlated to TNF-α and IL-1β levels, suggesting that oxidative stress was associated with various inflammatory factors in lung tissue. Previous study had demonstrated that oxidative stress could induce the expression of a variety of inflammatory cytokines by activating nuclear factor-κB (NF-κB) in cancers [35, 36]. In addition, in lung injury, lung cells could release inflammatory mediators and cytokines such as TNF-α, IL-1, and IL-8 in response to oxidative stress [7]. Therefore, these results prompted that the resistance mechanisms initiated by HSS in ALI were associated with the ability of HSS to inhibit oxidative stress and the synthesis of various inflammatory factors in lung tissue.

However, our study had some limitations. First, HSS was administered 5 min after OA treatment and OA-induced ALI might not be established at this time. So, in this study, we only can determine the protective effect but not the therapeutic effect, and we will design further experiments to address HSS post-injury treatment for ALI. Second, we did not detect hydrogen levels in the lungs, which might consolidate our present conclusions. Third, a control group receiving only HSS was missing in this study. Actually, a previous study has demonstrated that HSS cannot influence the levels of TNF-α, IL–6, MDA, and MPO in normal rats [37]. Thus, considering the constraints of research funding, the control group receiving only HSS was not set in this study. However, future comparative studies will be necessary to ascertain whether the approach advocated in the present investigation is really beneficial. Fourth, future studies are necessary to clarify the signaling pathways of this protection role provided by hydrogen. Lastly, more in vitro data are necessary to explore a mechanism of action in our future study. Despite these limitations, this preliminary study also could reveal the inhibitory effect of HSS on OA-induced ALI to some extent.

## Conclusion

To sum up, HSS attenuated OA-induced ALI in rats, and the delivery of HSS by ip injection was more effective than that by iv injection. This protective effect might be due to hydrogen’s ability to confer selective oxidation resistance and inhibit inflammatory infiltration in vivo. Thus, hydrogen had the potential to become an effective drug for the prevention of ALI.

## Abbreviations

ALI: Acute lung injury
BALF: Bronchial alveolar lavage fluid
ELISA: Enzyme-linked immunosorbent assay
HE: Hematoxylin and eosin
HSS: Hydrogen-saturated saline
I/R: Ischemia-reperfusion
IL-1β: Interleukin 1β
MDA: Malondialdehyde
MPO: Myeloperoxidase
OA: Oleic acid
SD: Standard deviation
TNF-α: Tumor necrosis factor-α
